# Oral frailty and activities of daily living among community-dwelling older adults: the indirect association of nutritional status

**DOI:** 10.3389/fpubh.2026.1903010

**Published:** 2026-08-31

**Authors:** Wei Jiang, Yaoqun Zhou, Xiaotong Feng, Chengyang Xie, Ronghua Fang, Xiaofeng Xie

**Affiliations:** 1 General Practice Ward, International Medical Center Ward, General Practice Medical Center, West China Hospital, Sichuan University/West China College of Nursing, Sichuan University, Chengdu, China; 2 West China Hospital, Sichuan University/West China College of Nursing, Sichuan University, Chengdu, China

**Keywords:** activities of daily living, community-dwelling older adults, indirect association, nutritional status, oral frailty

## Abstract

**Background:**

The acceleration of global population aging has made functional decline and increased care demand among older adults a major public health concern. Activities of daily living (ADL) are key indicators of basic function in older adults. Therefore, identifying factors associated with ADL among older people is critically important. This study aimed to examine the relationship between oral frailty and ADL among community-dwelling older adults in Chengdu, China, and to explore the statistical indirect association involving nutritional status.

**Methods:**

This multicenter cross-sectional study (January to March 2024) enrolled adults aged ≥60 years through convenience sampling from 12 community health service centers in Chengdu, China. Oral frailty, nutritional status, and ADL were assessed using the Oral Frailty Index-8 (OFI-8), Mini Nutritional Assessment-Short Form (MNA-SF), and Barthel Index (BI), respectively. Associations were examined using Spearman’s correlation analysis, multiple linear regression, and upper-censored Tobit regression. The statistical indirect association involving nutritional status was estimated using bootstrapping.

**Results:**

Among the 401 participants, 78 (19.5%) had impaired ADL, 164 (40.9%) had oral frailty, and 48 (12.0%) were at risk of malnutrition. Oral frailty was weakly negatively correlated with nutritional status (rs = −0.157, *p* < 0.01) and ADL (rs = −0.294, *p* < 0.01); nutritional status was weakly positively correlated with ADL (rs = 0.119, *p* < 0.01). After covariate adjustment, oral frailty was negatively associated with nutritional status (B = −0.116, *p* = 0.001), and nutritional status was positively associated with ADL (B = 3.129, *p* = 0.006). The direct association between oral frailty and ADL remained negative and significant after nutritional status was included (B = −2.261, *p* = 0.012). The indirect association through nutritional status was significant but small in magnitude (B = −0.363, 95% bootstrap: −0.798 - -0.044).

**Conclusion:**

Among community-dwelling older adults recruited in Chengdu, China, greater oral frailty was associated with poorer nutritional status and lower ADL. Nutritional status showed a statistically significant but small indirect association between oral frailty and ADL. Given the cross-sectional design, the temporal sequence and causal relationships among these factors cannot be established. Prospective studies are needed to confirm these findings.

## 1 Introduction

The global population is aging at an unprecedented pace. The number of people aged 60 years and older worldwide is projected to increase from 1 billion in 2020 to 1.4 billion by 2030, and further to approximately 2.1 billion by 2050 (1). China is undergoing a similarly rapid and profound demographic transition. Currently, individuals aged 60 years and above account for 23% of the total population (2), a proportion projected to exceed 30% by 2035, when the older population is expected to surpass 400 million (3). Chengdu, a major city in western China, is also experiencing rapid population aging. According to the seventh national census, adults aged 60 years and above accounted for 17.98% of Chengdu’s permanent population in 2020, representing an increase of 3.08 percentage points compared with 2010 (4). As the older population expands, functional decline and escalating care demands have emerged as critical challenges for public health and social care systems (5).

Activities of daily living (ADL) are essential indicators of physical function in the aging population, reflecting the ability to independently perform basic self-care activities such as eating, dressing, toileting, transferring, and walking (6). ADL impairment is closely associated with greater burden of chronic disease (7), lower social participation (8), an increased risk of falls (9), and higher mortality among older adults (10). It is also common among community-dwelling older adults (11). In Singapore, the number of older individuals with ADL impairment is projected to increase from 31,738 in 2010 to 82,968 in 2030 (12), and 4.5–7.4 million adults aged ≥65 years are projected to have three or more ADL limitations in the United States by 2040 (13). In China, the number of older people with functional impairment has reached 52.7 million (14). Because ADL impairment is often difficult to reverse (14), early identification of associated factors is critical for delaying functional decline and reducing the burden (9).

Oral frailty refers to the cumulative decline in multidimensional oral functions, including tooth loss, reduced masticatory function, impaired swallowing, diminished oral hygiene behaviors, and decreased social engagement (15, 16). Beyond reflecting oral health problems, oral frailty is closely linked to overall health status and is associated with adverse outcomes such as malnutrition, frailty, chronic diseases, sarcopenia, and severe functional impairment (15). In recent years, oral frailty has drawn more attention in geriatrics and public health. Evidence suggests that impaired oral function may link to poorer ADL performance (15, 17). Meanwhile, ADL impairment may affect people’s attention to oral hygiene and relate to greater oral frailty (18). Thus, there may be a reciprocal association between oral frailty and ADL, but the underlying associations require further clarification.

Nutritional status may be involved in the association between oral frailty and ADL impairment, yet malnutrition among community-dwelling older adults is often overlooked (19). Globally, 31–40% of community-dwelling older adults are at risk of malnutrition (20), and the prevalence of moderate to severe nutritional risk in China has been reported to reach 48.6% (21). Previous studies have shown that older adults with oral frailty have a higher risk of malnutrition, with reported prevalence rates ranging from 20.40–85.96% (22, 23). This association may be related to tooth loss, masticatory muscle atrophy, reduced salivary secretion, diminished taste perception, and impaired swallowing function (16). These changes have been linked to lower dietary diversity and poorer nutritional intake (24). Meanwhile, nutritional status is closely associated with ADL in older adults. Naseer et al. (10) reported that malnourished community-dwelling older adults were twice as likely to develop ADL dependence compared with well-nourished counterparts. Wei et al. (21) observed that each one-point increase in nutritional risk score was associated with a 58% increase in the risk of functional impairment among community-dwelling older adults. Thus, nutritional status may be relevant to the association between oral frailty and ADL.

The International Classification of Functioning, Disability and Health (ICF) provides a well-established theoretical framework that emphasizes the dynamic interactions among body functions and structures, activities and participation, and personal and environmental factors (25). In the present study, oral frailty, including chewing difficulty, swallowing impairment, and tooth loss, was conceptualized as an impairment in body function, whereas ADL reflected activity limitations and functional independence. These constructs are consistent with the domains described in the ICF framework (26, 27). Within this framework, oral frailty, nutritional status, and ADL may be interrelated. Poorer oral function may be associated with restricted food choices and nutritional intake (24). In turn, nutritional problems may coexist with fatigue, reduced muscle strength, and impaired physical functioning, which are related to poorer ADL (28, 29). Therefore, nutritional status was included as a theoretically relevant factor in the association between oral frailty and ADL. Given the cross-sectional design, this framework should be understood as a conceptual basis for examining statistical associations among variables measured at the same time, rather than as evidence of a causal sequence.

Other mechanisms may also be involved. One previous study suggested that oral frailty may be associated with inflammatory responses involving biomarkers such as C-reactive protein and interleukin-6, which may be linked to lower muscle mass, weaker muscle strength, and poorer physical performance (16). However, assessment of sarcopenia and inflammatory status generally requires measurements of muscle strength, muscle mass, physical performance, and blood-based biomarkers, which are often not feasible in routine community settings (30, 31).

Despite documented associations among oral frailty, nutritional status, and ADL, respectively (10, 22), few studies have examined these three factors together among community-dwelling older adults in Chengdu, China. Accordingly, guided by the ICF framework, the present study aimed to examine: (1) the association between oral frailty and ADL; (2) the association between oral frailty and nutritional status; and (3) whether nutritional status was statistically involved in the association between oral frailty and ADL. Given the cross-sectional design, this study examined statistical associations rather than causal pathways.

## 2 Methods

### 2.1 Study participants

From January to March 2024, a multicenter cross-sectional survey was conducted across 12 community health service centers in Chengdu, Sichuan Province, using convenience sampling. The target population comprised community-dwelling older adults. Community-dwelling adults aged ≥60 years who were able to understand and cooperate with the survey, and residing in the community for at least six months were included. Those with severe mental disorders, dementia, cognitive impairment with communication difficulties, or severe visual/hearing impairment were excluded. To minimize procedural bias across centers, all interviewers received standardized training before data collection, with uniform inclusion criteria, questionnaires, protocols, and quality control measures applied across sites. This study adhered to the Helsinki Declaration and was approved by the Ethics Committee of West China Hospital, Sichuan University (Ethics Approval No. 20232206). All participants provided written informed consent. The sample size was estimated using the formula for a cross-sectional study: $n=\frac{Z_{\alpha /2}^{2}P(1-P)}{d^{2}}$ . Based on a previously reported ADL impairment prevalence of 29.0% among community-dwelling older people in China (32), with a two-sided α level of 0.05 and an allowable error of 5%, the minimum required sample size was calculated to be 317 participants. After allowing for a 10% nonresponse rate, at least 352 participants were required. A total of 410 participants were recruited, 9 questionnaires were excluded, including 4 with substantial missing data, 3 with logically inconsistent responses, and 2 in which the same response option was selected for nearly all items. Finally, 401 valid questionnaires were included in the analysis, yielding a valid response rate of 97.8%.

### 2.2 Demographic questionnaire

The research team developed a baseline information form based on literature review, expert consultation, and pilot testing, collecting data on sex, age, marital status (unmarried/divorced/widowed/married), education, career (contract/compile), income source (children/pension), chronic disease (yes/no), exercise intensity (never/1–2 times/≥3 times), and exercise type (never/aerobic/resistance/combined).

### 2.3 Data collection

Trained interviewers conducted on-site surveys at each community health center. Participants were informed of the study purpose, content, and voluntary nature before providing written consent. The survey was administered using paper-based questionnaires with standardized instructions. For older adults with limited education, reading difficulties, or visual impairment, interviewers provided one-on-one assistance without suggesting answers.

### 2.4 Oral frailty assessment

Oral frailty among community-dwelling older adults was screened using the 8-item Oral Frailty Index-8 (OFI-8) by Tanaka et al. (15), encompassing tooth loss, oral functional decline, oral health-related behaviors, and reduced social participation. Three core items reflecting key oral functional impairment—subjective chewing difficulty, swallowing difficulty, and tooth loss—are weighted 2 points each, with the remaining items scored 1 point each. The total score ranges from 0 to 11, with higher scores indicating greater oral frailty. Oral frailty was defined as an OFI-8 score ≥4; an OFI-8 score <4 indicated no oral frailty. The OFI-8 in Chinese demonstrates good reliability: Cronbach’s *α* = 0.949, test–retest reliability coefficient = 0.786 (33).

### 2.5 Nutritional status assessment

Malnutrition risk in older adults was rapidly screened by the Mini Nutritional Assessment-Short Form (MNA-SF) by Rubenstein et al. (34), which consists of 6 items including body mass index, recent weight loss, acute disease or psychological stress, mobility, neuropsychological problems, and food intake. Scores range from 0 to 14, with higher scores indicating better nutritional status: according to the recommended criteria, 12–14 indicates normal status, 8–11 indicates malnutrition risk, and 0–7 indicates malnutrition (35). In descriptive analyses, malnutrition risk was defined as an MNA-SF score < 12.

### 2.6 ADL assessment

ADL was assessed using the internationally recognized 10-item Barthel Index (BI) (6), evaluating feeding, bathing, grooming, dressing, bowel control, bladder control, toileting, bed-to-chair transfer, ambulation, and stair climbing. Scores range from 0 to 100, with higher scores indicating better ADL performance. 0–20 indicate complete dependence; 21–60, severe dependence; 61–90, moderate dependence; 91–99, mild dependence; and 100, complete independence (6). Because the categories of complete, severe, and moderate dependence included very few participants in this study, these categories were not analyzed separately to avoid unstable estimates caused by sparse data. Therefore, ADL impairment was defined as a BI score < 100, and a BI score of 100 indicated normal ADL (7).

### 2.7 Oral health-related quality of life assessment

Oral health-related quality of life was assessed using Slade’s Oral Health Impact Profile-14 (OHIP-14) (36), a 14-item scale covering functional limitation, physical pain, psychological discomfort, physical disability, psychological disability, social disability, and handicap. Each item is scored from 0 (“never”), 1 (“hardly ever”), 2 (“occasionally”), 3 (“fairly often”), and 4 (“very often”), with total scores ranging from 0 to 56; higher scores reflect poorer oral health-related quality of life. The OHIP-14 in Chinese demonstrates favorable psychometric properties, with a Cronbach’s *α* of 0.93 and corrected item-total correlation coefficients of 0.53–0.71 (37).

## 3 Statistical analysis

All statistical analyses were conducted in SPSS 30.0 (IBM Corp., Armonk, NY, USA) and R (4.5.2) software. Continuous variables were summarized as mean ± SD, whereas categorical variables were presented as frequencies and percentages. Baseline characteristics were compared according to ADL. For descriptive analyses and group comparisons, ADL was classified as normal (BI = 100) or impaired (BI < 100). Categorical variables were compared using the χ^2^ or Fisher’s exact test. The OHIP-14 score was analyzed using the Mann–Whitney U test. Spearman’s correlation analysis was used to examine the associations among oral frailty, nutritional status, and ADL. Multiple linear regression analysis was used to estimate the association between oral frailty (OFI-8 score) and nutritional status (MNA-SF score). In correlation, regression, and indirect association analyses, the total MNA-SF score was treated as a continuous variable. Because the BI exhibited a substantial ceiling effect, with 323 participants (80.5%) achieving the maximum score of 100, upper-censored Tobit regression with an upper censoring point of 100 was used whenever the BI score was treated as the dependent variable.

Upper-censored Tobit regression was used to examine the associations of oral frailty and nutritional status with ADL, with ADL assessed by the BI score as the outcome variable. Oral frailty, assessed by the OFI-8 score, was first entered into the Tobit model to estimate its total association with ADL. Nutritional status, assessed by the MNA-SF score, and oral frailty were then entered simultaneously into the same Tobit model to estimate the adjusted association between oral frailty and ADL after accounting for nutritional status. This model was regarded as the primary analysis for examining the adjusted associations with ADL. The statistical indirect association involving nutritional status was examined as a secondary exploratory analysis using a product-of-coefficients approach. Nonparametric bootstrap resampling with 5,000 repetitions was used to estimate the total, direct, and indirect associations and their corresponding 95% bootstrap confidence intervals. All regression models were adjusted for sex, age, marital status, education, career, income source, chronic disease, exercise intensity, exercise type, and OHIP-14 score. Statistical significance was defined as a two-sided *p* value <0.05. In the bootstrap analysis, an association was considered statistically significant when its 95% bootstrap confidence interval did not include zero.

## 4 Results

### 4.1 Demographic characteristics

Of the 401 enrolled community-dwelling older adults (201 females, 50.1%), 175 (43.6%) were aged 60–70 years, 339 (84.5%) were married, 329 (82.0%) had formal education, 359 (89.5%) relied primarily on pension income, 266 (66.3%) had chronic diseases, and 65 (16.2%) never exercised. According to MNA-SF scores, 48 (12.0%) were at risk of malnutrition; 164 (40.9%) had oral frailty; 78 (19.5%) had ADL impairment; and the mean OHIP-14 score was 3.04 ± 4.91. Compared with participants with normal ADL, those with ADL impairment differed significantly in age, marital status, education, income source, chronic disease, weekly exercise frequency, exercise type, malnutrition risk, oral frailty, and OHIP-14 score (all *p* < 0.05), but not in sex or career (both *p* > 0.05) (Table 1).

**Table 1:** Demographic characteristics of community-dwelling older adults by ADL (BI) status (*N* = 401).

Variables	Total	ADL Normal (BI = 100, *n* = 323)	ADL Impairment (BI < 100, *n* = 78)	*p*
Sex				0.597
Male	200 (49.9)	159 (79.5)	41 (20.5)	
Female	201 (50.1)	164 (81.6)	37 (18.4)	
Age				<0.001
60 ~ 70	175 (43.6)	168 (96.0)	7 (4.0)	
71 ~ 80	157 (39.2)	124 (79.0)	33 (21.0)	
81 ~ 90	63 (15.7)	31 (49.2)	32 (50.8)	
≥91	6 (1.5)	0 (0.0)	6 (100.0)	
Marital status				0.038
Unmarried/divorced/widowed	62 (15.5)	44 (71.0)	18 (29.0)	
Married	339 (84.5)	279 (82.3)	60 (17.7)	
Education				<0.001
Yes	329 (82.0)	277 (84.2)	52 (15.8)	
No	72 (18.0)	46 (63.9)	26 (36.1)	
Career				0.694
Contract	344 (85.8)	276 (80.2)	68 (19.8)	
Compile	57 (14.2)	47 (82.5)	10 (17.5)	
Income source				0.005
Children	42 (10.5)	27 (64.3)	15 (35.7)	
Pension	359 (89.5)	296 (82.5)	63 (17.5)	
Chronic disease				<0.001
Yes	266 (66.3)	200 (75.2)	66 (24.8)	
No	135 (33.7)	123 (91.1)	12 (8.9)	
Exercise intensity weekly				<0.001
Never	65 (16.2)	41 (63.1)	24 (36.9)	
1–2 times	53 (13.2)	39 (73.6)	14 (26.4)	
≥3 times	283 (70.6)	243 (85.9)	40 (14.1)	
Exercise type				<0.001
Never	22 (5.5)	9 (40.9)	13 (59.1)	
Aerobic	348 (86.8)	286 (82.2)	62 (17.8)	
Resistance	1 (0.2)	1 (100.0)	0 (0.0)	
Aerobic and resistance	30 (7.5)	27 (90.0)	3 (10.0)	
Malnutrition				<0.001
Yes	48 (12.0)	29 (60.4)	19 (39.6)	
No	353 (88.0)	294 (83.3)	59 (16.7)	
Oral frailty				<0.001
Yes	164 (40.9)	115 (70.1)	49 (29.9)	
No	237 (59.1)	208 (87.8)	29 (12.2)	
OHIP-14 (M ± SD)	3.04 ± 4.91	2.69 ± 4.78	4.49 ± 5.21	0.005

OHIP-14, Oral Health Impact Profile-14; ADL = activities of daily living; BI = Barthel Index (higher score = better functional independence); OFI-8 = Oral Frailty Index-8 (higher score = greater oral frailty); MNA-SF = Mini Nutritional Assessment-Short Form (higher score = better nutritional status). Oral frailty was defined as OFI-8 ≥ 4, <4 = no. For descriptive analysis, the original three MNA-SF categories were collapsed into a binary variable, with MNA-SF <12 classified as at risk of malnutrition, ≥12 classified as not at risk of malnutrition.

### 4.2 Correlation analysis of oral frailty (OFI-8), nutritional status (MNA-SF), and ADL (BI)

Spearman correlation analysis showed that oral frailty was weakly negatively correlated with nutritional status (rs = −0.157, *p* < 0.01) and ADL (rs = −0.294, *p* < 0.01). Nutritional status was weakly positively correlated with ADL (rs = 0.119, *p* < 0.01) (Table 2).

**Table 2:** Spearman correlation analysis of oral frailty (OFI-8), nutritional status (MNA-SF), and ADL (BI).

Variable	Oral frailty (OFI-8)	Nutritional status (MNA-SF)	ADL (BI)
Oral frailty (OFI-8)	—		
Nutritional status (MNA-SF)	−0.157**	—	
ADL (BI)	−0.294**	0.119**	—

**p < 0.01; Oral frailty (OFI-8; higher score = greater oral frailty); Nutritional status (MNA-SF; higher score = better nutritional status); ADL (BI; higher score = better functional independence).

### 4.3 Correlations between individual oral frailty (OFI-8) components and nutritional status (MNA-SF) and ADL (BI)

Analysis of individual OFI-8 components revealed that nutritional status was weakly and negatively correlated with recent choking on tea or soup (rs = −0.112, *p* < 0.05), reduced outdoor activities in the past year (rs = −0.191, *p* < 0.01), difficulty chewing hard foods (rs = −0.125, *p* < 0.05), and brushing teeth fewer than twice daily (rs = −0.146, *p* < 0.01). Correlation analysis between ADL and OFI-8 components showed that ADL was negatively correlated with difficulty swallowing hard food compared with 6 months ago (rs = −0.216), recent choking while drinking tea or soup (rs = −0.164), current use of dentures (rs = −0.143), frequent complaints of dry mouth (rs = −0.183), reduced outdoor activities over the past year (rs = −0.342), and difficulty chewing hard food (rs = −0.188) (all *p* < 0.01) (Supplementary Table 1).

### 4.4 Multivariable regression analysis of oral frailty (OFI-8), nutritional status (MNA-SF), and ADL (BI)

**Figure 1:**
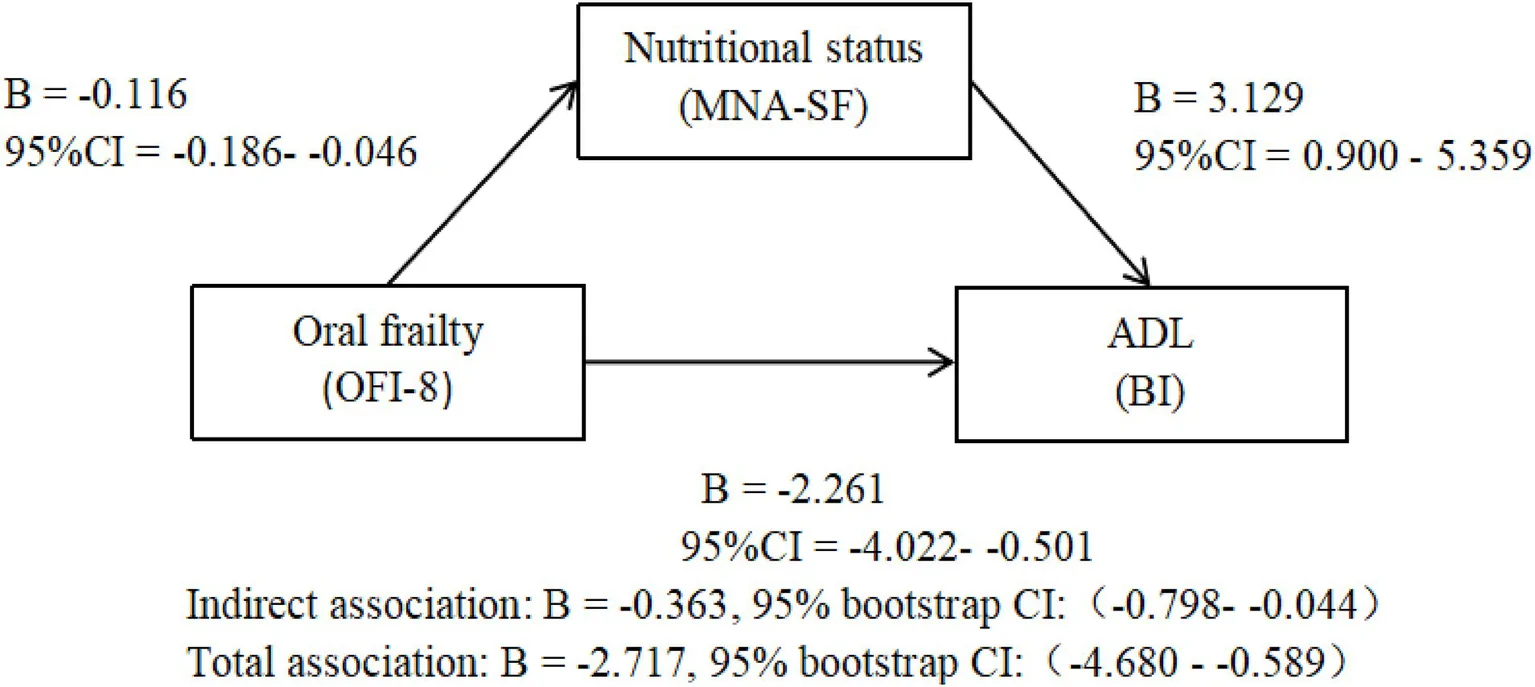
Statistical indirect association among oral frailty, nutritional status, and ADL. Nutritional status (MNA-SF; higher score, better nutritional status); Oral frailty (OFI-8; higher score, greater oral frailty); ADL (BI; higher score, better functional independence).

After adjustment for sex, age, marital status, education, career, income source, chronic disease, exercise intensity, exercise type, and the OHIP-14, the associations among oral frailty, nutritional status, and ADL were examined. Multiple linear regression analysis showed that oral frailty was negatively associated with nutritional status (B = −0.116, 95% CI: −0.186 - -0.046). Upper-censored Tobit regression showed that oral frailty was negatively associated with ADL before nutritional status was included in the model (B = −2.717, 95% CI: −4.528 - -0.905), when oral frailty and nutritional status were simultaneously included in the model, nutritional status was positively associated with ADL (B = 3.129, 95% CI: 0.900–5.359). Oral frailty remained negatively associated with ADL (B = −2.261, 95% CI: −4.022 - −0.501) (Figure 1; Table 3).

**Table 3:** Multivariable regression analysis of oral frailty (OFI-8), nutritional status (MNA-SF), and ADL (BI).

Predictor	Outcome variable	Statistical method	*B*	*SE*	*t*/*z*	95% LLCI	95% UL CI	*p*
Oral frailty (OFI-8)	Nutritional status	Multiple linear regression	−0.116	0.036	−3.236	−0.186	−0.046	0.001
Oral frailty (OFI-8)	ADL (BI)	Upper-censored Tobit regression	−2.717	0.924	−2.939	−4.528	−0.905	0.003
Nutritional status (MNA-SF)	ADL (BI)	Upper-censored Tobit regression	3.129	1.137	2.751	0.900	5.359	0.006
Oral frailty (OFI-8)	ADL (BI)	Upper-censored Tobit regression	−2.261	0.898	−2.517	−4.022	−0.501	0.012

B, unstandardized regression coefficient; 95% LLCI, lower limit confidence interval; 95% ULCI, upper limit confidence interval; Oral frailty (OFI-8; higher score = greater oral frailty); nutritional status (MNA-SF; higher score = better nutritional status); ADL (BI; higher score = better functional independence). The second row shows the association between oral frailty and ADL before nutritional status was included. In the fully adjusted Tobit model with ADL as the outcome, oral frailty and nutritional status were entered simultaneously as predictors. The fourth row shows the association between oral frailty and ADL after adjustment for nutritional status. All models were adjusted for sex, age, marital status, education, career, income source, chronic disease, exercise intensity, exercise type, and OHIP-14 score.

### 4.5 Secondary exploratory analysis of the statistical indirect association through nutritional status (MNA-SF)

Bootstrap analysis showed a statistically significant negative total association between oral frailty and ADL (B = −2.717, 95% bootstrap CI: −4.680- −0.589). The indirect association through nutritional status was negative but small in magnitude (B = −0.363, 95% bootstrap CI:-0.798 - −0.044), accounting for approximately 13.4% of the total association. After nutritional status was included in the model, a negative direct association between oral frailty and ADL remained significant (B = −2.261, 95% bootstrap CI: −4.100 - −0.188). The association model is presented in Figure 1, and the bootstrap estimates are shown in Table 4.

**Table 4:** Secondary exploratory analysis of the statistical indirect association through nutritional status (MNA-SF).

Association	Estimate	Boot SE	95% bootstrap LLCI	95% bootstrap ULCI	Proportion of total association
Total association	−2.717	1.033	−4.680	−0.589	
Direct association	−2.261	0.970	−4.100	−0.188	
Indirect association	−0.363	0.198	−0.798	−0.044	13.4%

## 5 Discussion

This study revealed that greater oral frailty was associated with poorer nutritional status and lower ADL performance, whereas better nutritional status was associated with higher ADL performance. However, the observed associations among oral frailty, nutritional status, and ADL were generally weak. The indirect association involving nutritional status accounted for approximately 13.4% of the total association, which may be partly attributable to the relatively good functional status of the participants. From the perspective of the ICF framework, ADL is associated with a broad range of physical, psychological, and social factors. Nutritional status should therefore be regarded as only one of several factors statistically.

### 5.1 ADL status among community-dwelling older adults

In the present study, the prevalence of ADL impairment was 19.5%, which was higher than the 6.14% prevalence of at least one ADL limitation reported among Korean older adults (38), but lower than the 61.2% reported in a Singapore study of pre-frail and frail community-dwelling older adults (39). These differences may be related to variations in age structure, sample source, health status, socioeconomic background, ADL assessment tools, and the cut-off criteria used to define ADL impairment.

Consistent with earlier reports (11, 40), age, marital status, education, income source, chronic disease, exercise frequency and type, and oral health-related quality of life were associated with ADL status. Advancing age is commonly accompanied by poorer muscle strength, impaired balance, cognitive decline, and a greater burden of chronic disease, all of which are associated with poorer daily functioning (29, 40). Older adults with a spouse, higher educational attainment, and stable income may have greater social support, better health literacy, and improved access to healthcare resources, factors that are generally associated with better functioning status (41, 42). Conversely, chronic disease and multimorbidity may coexist with reduced activity tolerance, fatigue, and poorer psychological well-being (7, 41). Moreover, regular physical activity may help preserve muscle strength, physical fitness, and balance (43–45).

### 5.2 Association between oral frailty and nutritional status

Oral frailty was weakly and inversely associated with nutritional status, which is consistent with the findings of Iwasaki et al. (23). Recent choking on tea or soup, reduced outdoor activities over the past year, difficulty chewing hard food, and brushing teeth fewer than twice daily were also associated with poorer nutritional status. These associations may partly reflect differences in food selection and dietary intake among older adults with poorer oral function. Chewing difficulty and swallowing discomfort have been associated with lower consumption of harder-to-chew foods, such as meat, nuts, vegetables, and fruits, as well as lower intake of protein, vitamins, dietary fiber, and micronutrients (24, 46). Moreover, low brushing frequency may indicate suboptimal oral hygiene and has been associated with dental caries and periodontitis (47). These oral conditions have also been associated with chewing discomfort, limited food choices, and lower nutrient intake among older adults (48). For older adults with oral health problems, these findings suggest that oral functional and nutritional assessments should be considered in future community-based intervention studies. Nevertheless, the correlation between oral frailty and nutritional status observed in this study was modest, indicating that oral frailty is only one of the many factors associated with nutritional status. Previous studies have shown that socioeconomic circumstances (49), chronic disease (50), medication use (50), appetite (50), depressive symptoms (51), and social or family support (52) may also be relevant factors. Future studies should further consider the combined effects of multiple factors on oral frailty and nutritional status among community-dwelling older adults.

### 5.3 Association between oral frailty and ADL

Oral frailty was negatively associated with ADL, consistent with Dibello et al. (53). In the exploratory component-level correlation analysis, several OFI-8 items, including swallowing difficulty, choking, denture use, dry mouth, reduced outdoor activities over the past year, and chewing difficulty, were negatively correlated with ADL (Supplementary Table 1). These correlations were not corrected for multiple comparisons and should therefore be interpreted as exploratory. The link between oral frailty and ADL impairment may involve complex biological, psychological, and social mechanisms. For example, an unbalanced diet that is predominantly carbohydrate-based and lacks sufficient protein, minerals, and vitamins may be associated with physical frailty, which may also affect long-term ADL (40, 54, 55). Moreover, denture use may reflect tooth loss, which may be related to communication difficulties (56), reduce social participation, and lower physical activity (57). Periodontal disease, a major cause of tooth loss in later life, is a chronic oral condition that has been associated with an increased risk of functional disability among older adults (58). In addition, reduced masticatory function has been associated with cognitive impairment in older adults, possibly through reduced sensory stimulation or changes in brain function, which may be relevant to ADL (59, 60). Thus, oral frailty may reflect not only oral functional decline but also poorer overall health reserve in older adults. For community-dwelling older adults at risk of oral frailty, oral functional assessment, chewing and swallowing training, oral hygiene guidance, physical activity promotion, and nutritional education may have potential value in supporting overall functional health (61).

### 5.4 Nutritional status as a potential indirect association between oral frailty and ADL

This study found a statistically significant but small indirect association between oral frailty and ADL involving nutritional status. This suggests that nutritional status may contribute to a limited degree, and that other factors are likely to be involved in most of the association.

From a mechanistic perspective, tooth loss, chewing difficulty, swallowing discomfort, and dry mouth may impair eating ability, restrict food choices, and reduce dietary intake. A 2-year longitudinal study showed that community-dwelling older adults with oral frailty at baseline had a higher subsequent risk of nutritional deterioration (24, 62). Poorer nutritional status has also been associated with lower muscle mass, muscle strength, gait speed, balance, and ADL performance (21, 28). which in turn associated with poorer ADL among older adults (29). Furthermore, a 9-year cohort study from the Japan Gerontological Evaluation Study showed that fewer teeth, chewing difficulty, dry mouth, and choking were associated with a higher subsequent risk of functional disability (63), while another prospective study indicated that lower maximum occlusal force was was associated with an increased risk of incident functional disability among older adults (64).

The association between oral frailty and ADL may involve factors beyond nutritional status. A cross-sectional study reported that poorer oral health was associated with lower systemic muscle mass, weaker grip strength, slower gait speed, and a higher likelihood of sarcopenia (65). These factors have been associated with poorer ADL among older adults (29). In addition, dental caries and periodontitis may contribute to chronic systemic inflammation and immune dysregulation, which may in turn be associated with cognitive impairment and reduced physical functioning (66, 67).

However, oral and systemic muscle mass and strength, inflammatory markers, and cognitive function were not directly assessed in the present study. More importantly, even if these factors had been measured, the cross-sectional design and simultaneous measurement of variables would still preclude inference about mechanistic or causal pathways. Therefore, these explanations should be regarded as hypothetical and require confirmation in future longitudinal and interventional studies. Taken together, the association between oral frailty and ADL may reflect multiple interrelated factors, including nutritional status (28), sarcopenia (29), chronic inflammation and cognitive function (66), and frailty (53).

From the perspective of community health management, these findings suggest the potential value of considering oral frailty, nutritional risk, and ADL limitations together in community assessments. Among community-dwelling older adults who are vulnerable to oral frailty, a combined assessment of oral function, nutritional risk, and ADL may help identify areas for future evaluation, including oral hygiene care, chewing and swallowing support, nutritional assessment, and physical activity promotion. Future longitudinal and interventional studies are needed to clarify the temporal relationships among these factors and to determine whether improvements in oral function and nutritional status can help prevent or delay deterioration in ADL.

### 5.5 Limitations

This study has several limitations. First, although a statistically significant indirect association between oral frailty and ADL through nutritional status was observed, reverse causality among these variables cannot be ruled out based on the present observational data. Future longitudinal and interventional studies are needed to clarify the temporal sequence and potential causal relationships among oral frailty, nutritional status, and ADL. Second, the BI exhibited a substantial ceiling effect. Although upper-censored Tobit regression was used to account for this distributional feature, the relatively small number of uncensored observations may have limited the precision and stability of the estimates. Future studies should assess the robustness of these findings using alternative outcome specifications. In addition, the sample size was estimated based on the expected prevalence of ADL impairment, and the statistical power for the bootstrap analysis was not specifically assessed. Future studies should consider sample size estimation based on the expected indirect association. Third, because convenience sampling was used, information on non-respondents was not available, and potential nonresponse bias could not be formally assessed. However, the recruitment of participants from multiple community healthcare settings with diverse demographic characteristics may partly mitigate concerns regarding representativeness. Future studies should use probability-based sampling methods and larger, multicenter samples from different regions of China to improve representativeness and generalizability, and should further incorporate moderating factors or additional potential indirect associations to better capture the complex processes related to ADL in community and healthcare settings. Fourth, although several potential covariates were adjusted for, sarcopenia, inflammatory markers, and mild cognitive impairment were not included, and these might affect the observed association. Future studies should include standardized assessments of these factors to more accurately characterize their relationships with oral frailty, nutritional status, and ADL.

## 6 Conclusion

In conclusion, greater oral frailty was associated with poorer nutritional status and lower ADL performance among community-dwelling older adults in Chengdu, China. Nutritional status showed a statistically significant but small indirect association. These findings suggest that oral frailty, nutritional risk, and ADL limitations may be considered together in community-based geriatric assessments to facilitate a more comprehensive understanding of older adults’ health and functional status.

## Data Availability

The raw data supporting the conclusions of this article will be made available by the authors, without undue reservation.
