# Supplementary material for: Oral frailty and activities of daily living among community-dwelling older adults: the indirect association of nutritional status
Source: Front Public Health. 2026 Aug 31;14:1903010. doi: 10.3389/fpubh.2026.1903010 (PMC13572234; doi:10.3389/fpubh.2026.1903010)
Supplement: Supplementary file 1 [file Table_1.docx]

Supplementary Material

# **1** Supplementary Data

**Supplementary table 1: Spearman correlation coefficients between oral frailty components and nutrition, ADL.**

| **Variable** | **Item1** | **Item2** | **Item3** | **Item4** | **Item5** | **Item6** | **Item7** | **Item8** | **Nutrition** | **ADL** |
| --- | --- | --- | --- | --- | --- | --- | --- | --- | --- | --- |
| Item1 | — |  |  |  |  |  |  |  |  |  |
| Item2 | 0.323** | — |  |  |  |  |  |  |  |  |
| Item3 | 0.092 | 0.049 | — |  |  |  |  |  |  |  |
| Item4 | 0.411** | 0.375** | 0.106* | — |  |  |  |  |  |  |
| Item5 | 0.288** | 0.161** | 0.048 | 0.280** | — |  |  |  |  |  |
| Item6 | 0.380** | 0.201** | 0.074 | 0.206** | 0.207** | — |  |  |  |  |
| Item7 | 0.141** | 0.101* | -0.039 | 0.054 | 0.105* | 0.276** | — |  |  |  |
| Item8 | -0.062 | 0.001 | -.200** | 0.013 | 0.019 | 0.023 | 0.098 | — |  |  |
| Nutrition | -0.056 | -0.112* | -0.069 | -0.061 | -0.191** | -0.125* | -0.146** | -0.075 | — |  |
| ADL | -0.216** | -0.164** | -0.143** | -0.183** | -0.342** | -0.188** | -0.081 | 0.013 | 0.119* | — |

**Note**: Item 1 = Difficulty swallowing hard food compared to six months ago; Item 2 = Choking or coughing when drinking tea or soup recently; Item 3 = Current denture use; Item 4 = Frequent complaints of dry mouth; Item 5 = Reduced outdoor activities in the past year; Item 6 = Difficulty chewing hard food, such as hard radish and squid; Item 7 = Brushing teeth less than twice daily; Item 8 = Not receiving annual dental check-ups. **p*＜0.05，***p*＜0.01
